# Comment on “Renail failure suppresses muscle irisin expression, and irisin blunts cortical bone loss in mice” by Kawao et al. ‐ the authors' reply

**DOI:** 10.1002/jcsm.13135

**Published:** 2022-11-30

**Authors:** Naoyuki Kawao, Miku Kawaguchi, Takashi Ohira, Hiroki Ehara, Yuya Mizukami, Yoshimasa Takafuji, Hiroshi Kaji

**Affiliations:** ^1^ Department of Physiology and Regenerative Medicine Kindai University Faculty of Medicine Ohnohigashi, Osakasayama Osaka Japan

We would like to thank Drs. Bao, Hu and Li for the critical appraisal^1^ of our findings.^2^ In this study, we examined the effects of renal failure on muscle, bone and myokine expression as well as irisin treatment on renal failure‐induced muscle atrophy and cortical bone loss using female C57BL/6J mice with 5/6 nephrectomy (Nx). We then showed that renal failure reduced irisin expression in muscle tissues. Moreover, irisin treatment blunted cortical bone loss induced by renal failure in mice.

We agree that C57BL/6 mice are relatively resistant to 5/6 Nx‐induced chronic kidney disease (CKD), when compared with other strains.^3^, ^4^, ^5^ On the other hand, several previous studies showed that 5/6 Nx induces CKD‐related pathophysiology, including skeletal myopthy^6^, ^7^ and diminished cortical/trabecular bone microarchitecture^8^, ^9^ in C57BL/6 mice, although we have not shown the data of bone pathological features in our study. Moreover, 5/6 Nx induced renal fibrosis and decreased glomerular filtration rate in C57BL/6J mice.^10^, ^11^ We therefore believe that C57BL/6 mice could be used as a model to exhibit muscle atrophy and bone loss with the progression of CKD after 5/6 Nx. We would like to plan the study to examine myokine expression using the other CKD model mice in the near future.

Kim et al. recently have shown that Nx‐induced muscle atrophy was blunted in female C57BL/6J mice (6%–13% reduction), compared with male (18%–24% reduction), suggesting that there might be sex differences of muscle atrophy in C57BL/6J mice after 5/6 Nx.^6^ Nonetheless, we showed that 5/6 Nx elevated serum levels of blood urea nitrogen, creatinine and parathyroid hormone in female C57BL/6J mice.^2^ Moreover, 5/6 Nx decreased body weight, muscle mass in the whole body and lower limbs assessed by quantitative computed tomography, tissue weights of skeletal muscles and cortical bone mineral density at the femurs in female C57BL/6J mice.^2^ Previous studies showed that 5/6 Nx decreased cortical thickness in female C57BL/6J mice,^8^, ^12^ which are consistent with our findings.^2^ However, 5/6 Nx did not affect expressions of protein degradation‐related genes in muscular tissues of female C57BL/6J mice in our study.^2^ Although its reason has still remained unclear in our study, Svensson et al. revealed that estradiol suppresses atrogin‐1 mRNA level in the gastrocnemius muscles of male C57BL/6J mice.^13^ We therefore speculate that oestrogen might affect muscle protein degradation in female mice. Although irisin treatment did not affect 5/6 Nx‐induced muscle atrophy of female C57BL/6J mice in our study, further studies will be required to clarify its effects on male mice with other genetic backgrounds.

## Conflict of interest

The authors declare that they have no conflict of interests.
